# Supplementary material for: Hybrid Models and Biological Model Reduction with PyDSTool
Source: PLoS Comput Biol. 2012 Aug 9;8(8):e1002628. doi: 10.1371/journal.pcbi.1002628 (PMC3415397; doi:10.1371/journal.pcbi.1002628)
Supplement: Text S4 — Complete source code for the PyDSTool package (version 0.88.120504). Includes API documentation and help files linking to web pages. This file is identical to the current public release on Sourceforge.net. (ZIP) [file pcbi.1002628.s004.zip › PyDSTool/html/PyDSTool.scipy_ode.IntegratorBase-class.html]

xml version="1.0" encoding="ascii"?


PyDSTool.scipy\_ode.IntegratorBase


| Home | Trees | Indices | Help | PyDSTool |
| --- | --- | --- | --- | --- |

Package PyDSTool ::
Module scipy\_ode ::
Class IntegratorBase

[hide private]

[frames] | no frames]

# Class IntegratorBase

source code

```
object --+
         |
        IntegratorBase
```

Known Subclasses:
:   - vode

---


|  |  |
| --- | --- |
| Instance Methods | [hide private] |

|  |  |
| --- | --- |
| reset(self, n, has\_jac)  Prepare integrator for call: allocate memory, set flags, etc. | source code |

|  |  |
| --- | --- |
| run(self, f, jac, y0, t0, t1, f\_params, jac\_params)  Integrate from t=t0 to t=t1 using y0 as an initial condition. | source code |

|  |  |
| --- | --- |
| step(self, f, jac, y0, t0, t1, f\_params, jac\_params)  Make one integration step and return (y1,t1). | source code |

|  |  |
| --- | --- |
| run\_relax(self, f, jac, y0, t0, t1, f\_params, jac\_params)  Integrate from t=t0 to t>=t1 and return (y1,t). | source code |

**Inherited from `object`**:
`__delattr__`,
`__getattribute__`,
`__hash__`,
`__init__`,
`__new__`,
`__reduce__`,
`__reduce_ex__`,
`__repr__`,
`__setattr__`,
`__str__`


|  |  |
| --- | --- |
| Class Variables | [hide private] |

runner = `None`

success = `None`

supports\_run\_relax = `None`

supports\_step = `None`

integrator\_classes = `[<class 'PyDSTool.scipy_ode.vode'>]`


|  |  |
| --- | --- |
| Properties | [hide private] |

**Inherited from `object`**:
`__class__`


|  |  |
| --- | --- |
| Method Details | [hide private] |

|  |  |
| --- | --- |
| reset(self, n, has\_jac) | source code |

Prepare integrator for call: allocate memory, set flags, etc. n -
number of equations. has\_jac - if user has supplied function for
evaluating Jacobian.

|  |  |
| --- | --- |
| run(self, f, jac, y0, t0, t1, f\_params, jac\_params) | source code |

Integrate from t=t0 to t=t1 using y0 as an initial condition. Return
2-tuple (y1,t1) where y1 is the result and t=t1 defines the stoppage
coordinate of the result.

  


| Home | Trees | Indices | Help | PyDSTool |
| --- | --- | --- | --- | --- |

|  |  |
| --- | --- |
| Generated by Epydoc 3.0.1 on Fri May 4 15:24:10 2012 | http://epydoc.sourceforge.net |
